# Transcriptional Profiling of Endocrine Cerebro-Osteodysplasia Using Microarray and Next-Generation Sequencing

**DOI:** 10.1371/journal.pone.0025400

**Published:** 2011-09-27

**Authors:** Piya Lahiry, Leo J. Lee, Brendan J. Frey, C. Anthony Rupar, Victoria M. Siu, Benjamin J. Blencowe, Robert A. Hegele

**Affiliations:** 1 Robarts Research Institute, London, Ontario, Canada; 2 Department of Medicine, Schulich School of Medicine and Dentistry, University of Western Ontario, London, Ontario, Canada; 3 Department of Biochemistry, Schulich School of Medicine and Dentistry, University of Western Ontario, London, Ontario, Canada; 4 Department of Pediatrics, Schulich School of Medicine and Dentistry, University of Western Ontario, London, Ontario, Canada; 5 Banting and Best Department of Medical Research and Department of Molecular Genetics, University of Toronto, Toronto, Ontario, Canada; 6 Department of Electrical & Computer Engineering, University of Toronto, Toronto, Ontario, Canada; 7 Children's Health Research Institute, Lawson Health Research Institute, London, Ontario, Canada; Massachusetts General Hospital and Harvard Medical School, United States of America

## Abstract

**Background:**

Transcriptome profiling of patterns of RNA expression is a powerful approach to identify networks of genes that play a role in disease. To date, most mRNA profiling of tissues has been accomplished using microarrays, but next-generation sequencing can offer a richer and more comprehensive picture.

**Methodology/Principal Findings:**

ECO is a rare multi-system developmental disorder caused by a homozygous mutation in *ICK* encoding intestinal cell kinase. We performed gene expression profiling using both cDNA microarrays and next-generation mRNA sequencing (mRNA-seq) of skin fibroblasts from ECO-affected subjects. We then validated a subset of differentially expressed transcripts identified by each method using quantitative reverse transcription-polymerase chain reaction (qRT-PCR). Finally, we used gene ontology (GO) to identify critical pathways and processes that were abnormal according to each technical platform. Methodologically, mRNA-seq identifies a much larger number of differentially expressed genes with much better correlation to qRT-PCR results than the microarray (r^2^ = 0.794 and 0.137, respectively). Biologically, cDNA microarray identified functional pathways focused on anatomical structure and development, while the mRNA-seq platform identified a higher proportion of genes involved in cell division and DNA replication pathways.

**Conclusions/Significance:**

Transcriptome profiling with mRNA-seq had greater sensitivity, range and accuracy than the microarray. The two platforms generated different but complementary hypotheses for further evaluation.

## Introduction

New technologies permit the evaluation of global patterns of gene expression – mRNA levels – from healthy and diseased tissues. The simultaneous assessment of changes in expression of many genes – up to the whole genome level – can then be analysed simultaneously using bioinformatic tools that can reveal new patterns or networks of differentially regulated genes [1]. These technologies have transformed our conception of the molecular mechanisms underlying complex diseases such as cancer and degenerative illnesses [2], [3]. Over the past five years, microarrays – which are a hybridization-based technology – have been the main platform used for transcription profiling. However, within the last two years, high throughput next-generation mRNA sequencing methods now allow for quantitative measurement of expression levels on a genome-wide basis at the level of a single nucleotide.

We had the opportunity to compare technologies used to generate expression profiles of cultured fibroblasts from Amish children with a rare autosomal recessive condition called endocrine-cerebro-osteodysplasia (ECO; MIM 612651). ECO is a multi-system neonatal lethal disorder – a kinasopathy [4] – affecting mainly the skeletal, cerebral and endocrine systems that results from a homozygous nonsynonymous mutation (R272Q) in the *ICK* gene encoding intestinal cell kinase [5]. ICK, also known as MAK-related kinase (MRK), is ubiquitously expressed, particularly in brain, spinal cord, testis, and ovary [6], [7], [8]. Catalytic domains of ICK share ∼40% identity with those of consensus MAPKs, which are regulators of cell-cycle entry and transition by cyclin-dependent protein kinases (CDKs) [9]. Residue 272 lies within the nuclear localization signal sequence [5], [9] and the R272Q mutation both impairs nuclear localization and reduces catalytic activity [5].

Since very little was known about the downstream biological pathways and gene networks that were affected in ECO patients, we profiled the transcriptomes of cultured skin fibroblasts from ECO patients. We used 2 independent technological platforms to accomplish this, namely cDNA microarrays and next-generation mRNA sequencing (mRNA-seq). This provided a unique opportunity to validate the findings of each platform using quantitative RT-PCR (qRT-PCR) and to compare the networks of genes that were identified by GOStat, a database that lists all the overrepresented GO terms according to statistical significance [10], and KEGG pathway, a collection of manually curated pathway maps [11].

## Materials and Methods

### Participants and Ethics Statement

Primary skin fibroblasts from two subjects affected with ECO (designated 030950 and 040786) and one unrelated unaffected from the community (070280) were obtained from forearm puncture biopsies from affected individuals. The skin fibroblast line AG03348 (or 3348) was obtained from the Coriell Cell Repository (Coriell Institute for Medical Research, Camden NJ), and served as another unaffected non-Amish control cell line. Cultured primary skin fibroblasts were maintained at 37°C and 5% CO_2_ in Ham's F-10 medium (Gibco, Carlsbad CA) with L-glutamine supplemented with 10% fetal bovine serum and 1× antibiotic/antimycotic mixture (Gibco). For passaging, cells were released from the dish using 0.1% (w/v) trypsin and 0.02% (w/v) EDTA washes and re-distributed onto another dish. Samples from all passages were stored in −80°C.

Tissue samples were provided for research purposes, with approval by the Office of Research Ethics (University of Western Ontario). Participating parents provided informed consents and did not receive any financial compensation.

### Skin Fibroblast Cell Line Doubling Measurements

Each cell line was passaged and maintained in 90 mm diameter dishes (Gibco) two to three times weekly. After release using trypsin and EDTA, washed cells were diluted in enriched Ham's F-10 medium. Ten microlitres of re-diluted cells were counted using a haemocytometer and seeded on fresh culture dishes. Cell number counted (n) was used to calculate the number of cells per mL (N), with the formula N = n×10^4^. This procedure was carried out until the same number of cells or fewer was obtained from sub-culturing over three consecutive passages. Cell growth was measured by calculation of population doubling (PD) using the formula:
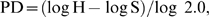
where log H is the logarithm of the number of cells harvested after 3 or 4 days of growth and log S is the logarithm of the number of cells on the first day of each passage. Accumulated population doublings (APD) were calculated by the summation of PDs.

### RNA Isolation

For each cell line, RNA from a “young” cultured age passage was extracted at an APD of 3–5 whereas RNA from an “old” cultured age passage was extracted at an APD of ∼20–22. In total 8 samples were extracted, with young and old passages for affected cell lines 030950 and 040786 and for unaffected cell lines 070280 and 3348. RNA was extracted from cultured skin fibroblasts using the RNEasy kit (Qiagen, Mississauga, ON). Briefly, cells were lysed with a buffer containing guanidine-isothiocyanate and β-mercaptoehtanol. Genomic DNA was sheared using a Shredder column (Qiagen). Ethanol was added to the resulting solution allowing the RNA to bind to the silicia-gel-membrane spin column. Bound RNA was washed with ethanol and eluted with RNAse-free water. Once RNA was extracted, its concentration and purity were measured using the NanoDrop spectrophotometer (Thermo Scientific; Ottawa, ON) and the Agilent 2100 Bioanalyzer (Agilent Technologies; Palo Alto, CA). Samples were stored at −80°C.

### cDNA Microarray Hybridization and Analysis

All microarrays were processed at the London Regional Genomics Centre (http://www.lrgc.ca). Biotinylated RNA was prepared from 2 µg of total RNA using the two–cycle amplification protocol. Double-stranded cDNA was synthesized using SuperScript II (Invitrogen, Carlsbad, CA) and oligonucleotide primers. Biotin-labelled complementary RNA (cRNA) with incorporated biotinylated UTP and CTP was prepared using *in vitro* transcription of cDNA with the Bizarre High-Yield RNA Transcript Labeling kit (Enzo Brioche, New York, NY). Fifteen micrograms of labelled cRNA was hybridized to Human 1.0 ST array GeneChips for 16 h at 45°C (Affymetrix, Santa Clara, CA). The chips were stained with streptavidin-phycoerythrin solution. Liquid handling was performed by the GeneChip Fluidics Station 450 (Affymetrix) and arrays were scanned using the GeneChip Scanner 3000 (Affymetrix). Signal intensities for genes were generated using GCOS1.4 (Affymetrix) using default values for the Statistical Expression algorithm parameters. Probe level data was imported into Genomics Suite software (Partek, St. Louis, MO); the student's paired *t* test was used to detect differences between them.

### mRNA Deep Sequencing Platform Hybridization and Analysis

Five µg of total RNA was processed using proprietary kits from Illumina (Hayward, CA). Briefly, PolyA^+^ RNA was isolated from total RNA fragmented using Ambion RNA fragmentation buffer. cDNA synthesis was performed with Invitrogen random hexamer primers and cDNA was purified using QIAquick PCR spin column (Qiagen). Ends were blunted and 3′-A overhangs introduced using T4 DNA polymerase and *E. coli* DNA polymerase I Klenow fragment. cDNAs were ligated to adapters with a single ‘T’ base overhang. After selection of 150–200 bp fragments from 2% low-range agarose gel, samples were amplified by 18 PCR cycles to enrich cDNAs with correctly ligated adapters and to amplify the amount of DNA in the library. Samples were loaded on a Cluster Station to create flow cells of clonal single molecular array (CSMA) and sequenced on the Illumina platform [12]. The analysis pipeline encompassed primary data acquisition, base calling, and calculating confidence scores from the fluorescence signals on the Genome Analyzer. Each transcriptome was sequenced at a depth of 30–40 million single reads, with read lengths up to 75 bp. Raw reads were converted to FASTQ data format since this format compactly stores a quality score for each base, which could be used to filter individual sequences. The quality-filtered reads were then aligned by TopHat [13], which map them to both the UCSC reference human genome and exon-exon splice junctions as annotated by Ensembl. Cufflinks [14] then provided the gene expression levels, based on the TopHat alignments and Ensembl annotation. Gene expression was quantified as ‘reads per kilobase of exon model per million mapped reads’ (RPKM) [15], and the expression cutoff was 0.5 RPKM — that is, the transcript of the gene was present if there were ≥10 reads that mapped uniquely to a single genomic locus. More than 18,815 Ensembl annotated protein-coding genes were compared to create a gene list of differentially expressed genes based on disease status of the cell lines. Transcript level data were then imported into Genomics Suite (Partek, St. Louis, MO) for additional analyses; comparisons were performed using student's paired *t* test.

### Quantitative RT-PCR

For the mRNA-seq platform gene list, two probes per gene were chosen for *FBLN5*, *EMP1*, *CHPF*, *EXT1*, *CRIP1*, *MEST*, *STC2*, *AFAP1*, *DKK2*, *LRRK2*, *LXN*, *FAM20A*, *DYNC1I1*, *KIF23*, and *GPR160*, while one probe was chosen for *SOD3, RAP1B, CCRL1, and HTR1B* based on probe availability (Gene Expression Assay, Applied Biosystems, Carlsbad, CA). qRT-PCR standard curves for *FBLN5*, *EMP1*, *SOD3*, *CHPF*, *CRIP1*, *DKK2*, *LRRK2*, *CCRL1*, *FAM20A*, and *DYNC1I1* were acquired using cell line 070280, while cell line 030950 was used to derive standard curves for *EXT1*, *MEST*, *STC2*, *RAP1B*, *AFAP1*, *LXN*, *KIF23*, *GPR160*, and *HTR1B*.

Total RNA (100 ng) was reverse transcribed using the High-Capacity cDNA Reverse Transcription Kit (Applied Biosystems) in a 20 µL reaction volume and amplified using TaqMan Assay probes (Applied Biosystems) in a 7900 HT Real Time PCR System (Applied Biosystems) with the 40 cycle amplification protocol. Amplified sequences were detected using the Prism sequence detector (Applied Biosystems) according to manufacturer's instructions. Experiments were done in triplicate, using *GAPDH* as an internal reference, on the young and old age passages from affected cell lines 030950 and 040786 and from unaffected cell lines 070280 and 3348. Expression values were standardized to values obtained with the standard RNA using the delta Ct method. Standard curves had r^2^ values>0.98.

### Biological Interpretations

The cDNA microarray data set was first run on the gene ontology (GO)-ANOVA analysis tool (Partek). The mRNA-seq data set was also biologically interpreted using GO through a web-based tool, GOStat (http://gostat.wehi.edu.au/), which finds statistically overrepresented GO terms within the provided data set. In addition, gene lists based on disease status from the microarray and the RNA-seq platforms were analyzed by Pathway Express (http://vortex.cs.wayne.edu/projects.htm), which uses the KEGG pathway database to define biological and cellular functions.

## Results

### Hyperproliferation of Cultured Skin Fibroblasts from ECO-Affected Individuals

**Figure 1:**
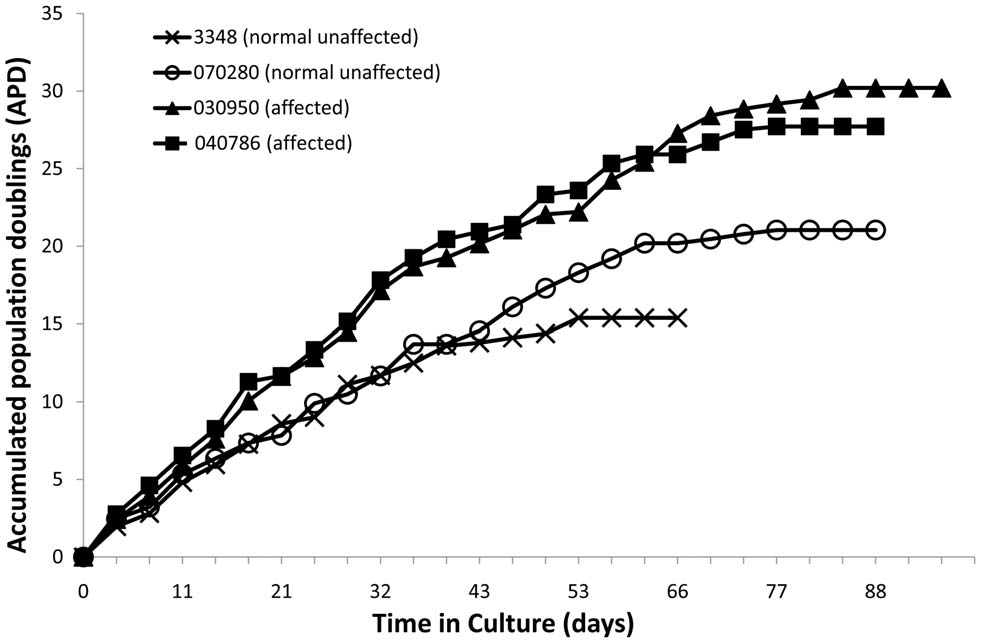
Growth characteristics of skin fibroblasts from ECO-affected and unaffected individuals. The accumulated population doublings (y-axis) achieved at indicated time in culture (x-axis) for four fibroblast cell lines. The fibroblast cell lines include: two homozygous normal subjects (070280 and 3348) and the two homozygous affected subjects (030950 and 040786).

The complete lifespan growth curves of the cell lines are shown (Figure 1). To reach senescence, the 030950 cell line was passaged 27 times for 96 days, 040786 was passaged 25 times for 88 days, 070280 was passaged 25 times for 88 days, and 3348 was passaged 19 times for 66 days. The mean PD from the 2 normal fibroblast lines was 0.59±0.03, while the mean PD for the 2 ECO-affected fibroblast cell lines was 1.07±0.05 (P<0.05). These findings, obtained from experiments performed in duplicate, indicated an approximate doubling of the proliferation rate in affected skin fibroblasts compared to unaffected skin fibroblasts.

### Transcriptome Profiling

Both microarray and mRNA-seq platforms showed consistently high numbers of genes that were differentially expressed in ECO-affected versus unaffected fibroblasts. Table 1 shows a comparison of the number of significantly differentially expressed genes based on fold change determined by each platform. Overall, mRNA-seq identified a greater number of differentially expressed genes with fold changes ≥2.0 than microarrays (708 versus 206, respectively), with virtually identical numbers of genes with fold changes between 1.0 and 2.0 (1636 versus 1592, respectively) indicating that mRNA-seq was more sensitive in identifying significantly differentially expressed genes than the cDNA microarray.

**Table 1:** Fold change distribution of differentially expressed genes based on disease status using both platforms (P≤0.05).

	Platform	Fold change					
		≥30	≥20	≥10	≥5	≥2	≥1
Upregulated genes in ECO-Affected	cDNA microarray	0	0	2	6	62	618
	mRNA-seq	11	16	42	99	453	1179
Downregulated genes in ECO-Affected	cDNA microarray	0	1	3	11	144	974
	mRNA-seq	5	7	29	74	255	457
**Total differentially expressed genes**	**cDNA microarray**	**0**	**1**	**5**	**17**	**206**	**1592**
	**mRNA-seq**	**16**	**23**	**71**	**173**	**708**	**1636**

The cDNA microarray identified more downregulated genes, while the mRNA-seq platform identified more upregulated genes. Most differentially expressed genes from the cDNA microarray had a fold-change range from 2 to 10, while the differentially expressed genes from the mRNA-seq platform had a greater range in fold-change values, and many more genes with fold changes ≥20.0.

**Figure 2:**
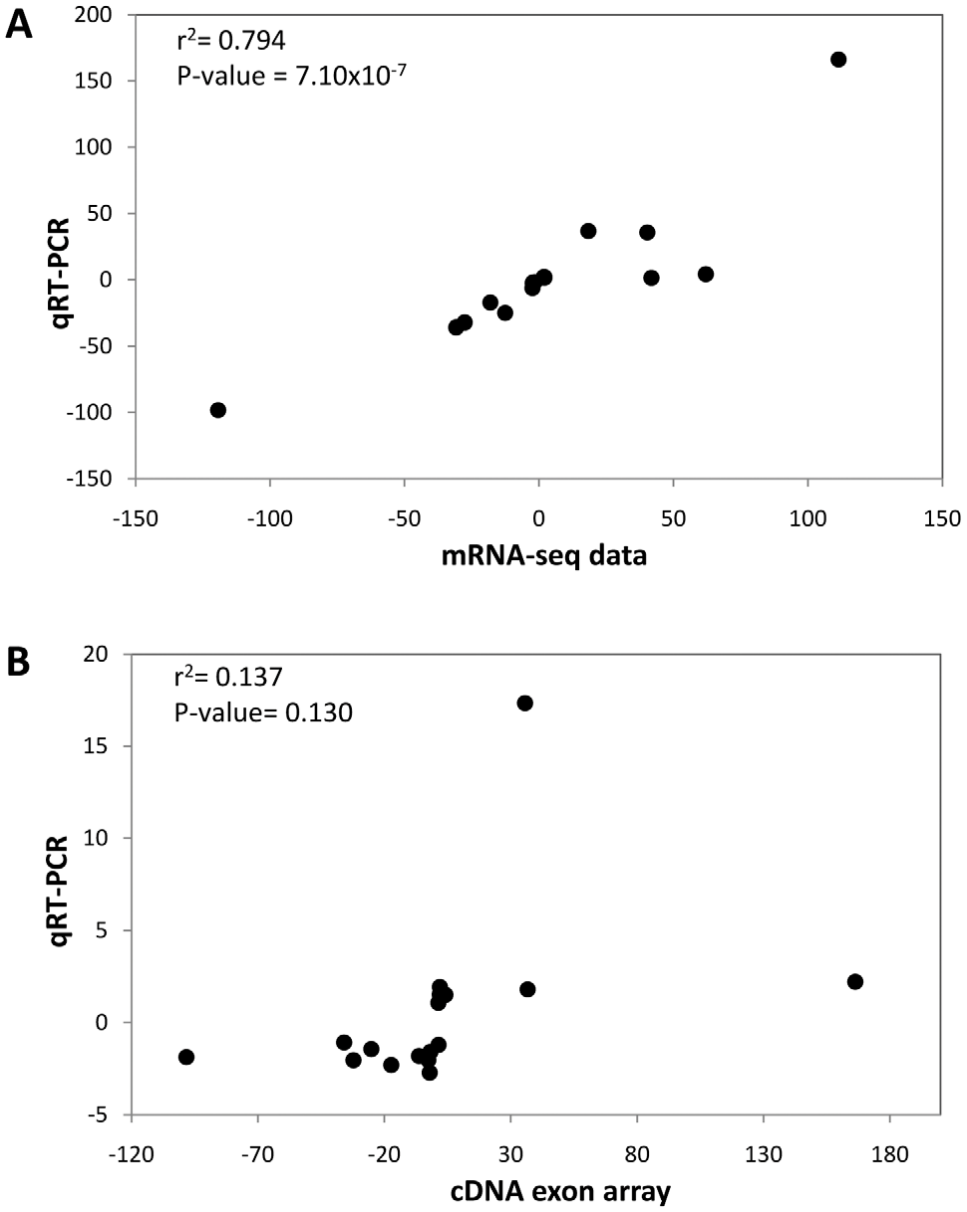
Correlation graphs of the fold change of the 18 genes selected from the mRNA-seq platform. (A) The fold-change values from the mRNA-seq data (x-axis) are plotted against the fold change value from the qRT-PCR experiments (y-axis), giving good correlation (r^2^ = 0.794, P = 7.10×10^−7^). (B) The fold-change values from the cDNA microarray data (x-axis) are plotted against the fold change value from the qRT-PCR experiments (y-axis), with non-significant correlation (r^2^ = 0.137, NS).

We next validated 20 significant differentially expressed genes from the mRNA-seq platform using qRT-PCR. Candidate genes were chosen based on conventional criteria [16] such as >2-fold change between conditions, with P≤0.05, regardless of the ‘age’ or passage number (see Table 2). By inspection, the direction and degree of fold- changes were more similar to the qRT-PCR findings for mRNA-seq identified genes than for microarray identified genes. Also, by inspection, there appeared to be systematic underestimation in fold-change values from the cDNA microarray data set, for about half of the validation gene set, namely *SOD3*, *CRIP1*, *MEST*, *DKK2*, *LXN*, *CCRL1*, *FAM20A*, *DYNC1I1*, *HTR1B*, and *RASGRP1*. Also, 5 genes, namely *STC2*, *RAP1B*, *DYNC1I1*, *KIF23* and *GPR160* were not statistically different in terms of gene expression between the platforms. There was much better correlation between the mRNA-seq platform and qRT-PCR values (r^2^ = 0.794, P = 7.10×10^−7^, Figure 2A) than between the cDNA microarray and qRT-PCR (r^2^ = 0.137, P = 0.12, Figure 2B).

**Table 2:** Comparison of fold change values of 20 genes (with P≤0.05) selected from the mRNA-seq platform to qRT-PCR and cDNA microarray.

Gene symbol	Gene Name	mRNA-seq data (P≤0.05)			qRT-PCR (Fold change)	cDNA microarray (Fold change)
		Affected^a^	Unaffected^a^	Fold change		
*AFAP1*	Actin filament associated protein 1	75.1	37.5	2.00	1.97	1.94
*CCRL1* b	Chemokine (C-C motif) receptor-like 1	0.19	22.8	−119.3	−98.3	−1.87
*CHPF*	Chondroitin polymerizing factor	142.3	231.9	−1.63	−1.80	−1.59
*CRIP1*	Cysteine-rich intestinal protein 1	39.0	95.0	−2.44	−6.32	−1.81
*DKK2*	Dickkopf homolog 2	0.16	2.81	−18.1	−17.3	−2.30
*DYNC1I1*	Dynein, cytoplasmic 1, intermediate chain 1	0.02	0.56	−30.8	−35.9	−1.08^c^
*EMP1*	Epithelial membrane protein 1	99.4	217.2	−2.18	−2.06	−2.72
*EXT1*	Exostosin 1	184.3	104.3	1.77	1.95	1.55
*FAM20A*	Family with sequence similarity 20, member A	0.27	7.48	−27.7	−32.2	−2.04
*FBLN5*	Fibulin-5	73.1	172.8	−2.36	−2.60	−2.03
*GPR160*	G protein-coupled receptor 160	0.78	0.02	41.8	1.37	1.08^c^
*HTR1B* b	5-hydroxytryptamine (serotonin) receptor 1B	0.87	0.01	62.0	4.15	1.51
*KIF23*	Kinesin family member 23	601.9	2.03	295.9	1.17	1.30^c^
*LRRK2*	Leucine-rich repeat kinase 2	0.05	1.30	−27.1	−29.8	n/a
*LXN*	Latexin	10.7	0.10	111.4	166.3	2.22
*MEST*	Mesoderm-specific transcript homolog	83.6	4.56	18.3	36.7	1.80
*RAP1B* b	Ras-related protein 1b	78.9	39.1	2.02	1.46	−1.21^c^
*RASGRP1*	RAS guanyl releasing protein 1 (calcium and DAG-regulated)	19.1	0.47	40.3	35.7	17.3
*SOD3* b	Superoxide dismutase 3, extracellular	6.44	81.1	−12.6	−25.1	−1.44
*STC2*	Stanniocalcin 2	149.3	85.9	1.74	1.90	1.52^c^

a values based on RPKM normalization;

b one qRT-PCR probe used;

c not significant.

### Biological Interpretations of Differentially Expressed Genes

Using GOStat [10] we determined the top 20 overrepresented GO terms based on the total number of genes that were significantly (P<0.05) differentially expressed from microarray and mRNA-seq platforms (Tables 3 and 4, respectively). GO categories identified as significant by microarray tended towards anatomical and organ development and morphogenesis. In contrast, GO categories identified as significant by mRNA-seq data tended towards genes involved in cell cycling and cell division. Together, the findings suggest that differentially expressed genes in the ECO syndrome are found in pathways involved in the proliferation and regulation of cell cycle.

**Table 3:** Top 20 overrepresented Gene Ontology (GO) terms using GOStat in the cDNA microarray data set (based on disease status, P≤0.05 and ≥±2.0 fold change, 206 genes).

GO ID	GO category	P-value	# Genes/ GO ID
48856	anatomical structure development	5.84×10^−14^	39
7275	multicellular organismal development	8.70×10^−14^	42
32502	developmental process	6.80×10^−13^	52
32501	multicellular organismal process	5.57×10^−11^	54
48731	multicellular organismal system development	5.18×10^−10^	30
48513	organ development	2.00×10^−7^	22
8283	cell proliferation	2.13×10^−7^	21
7165	signal transduction	9.44×10^−7^	58
7154	cell communication	1.04×10^−6^	61
9653	anatomical structure morphogenesis	1.04×10^−6^	20
65007	biological regulation	1.75×10^−5^	67
65008	regulation of biological quality	7.38×10^−5^	17
48523	negative regulation of cellular process	7.38×10^−5^	19
48519	negative regulation of biological process	1.76×10^−4^	19
48869	cellular developmental process	2.16×10^−4^	25
30154	cell differentiation	2.16×10^−4^	25
50789	regulation of biological process	3.63×10^−4^	59
50794	regulation of cellular process	7.33×10^−4^	55
7259	JAK-STAT cascade	2.73×10^−3^	4
9605	response to external stimulus	2.73×10^−3^	13

**Table 4:** Top 20 overrepresented Gene Ontology (GO) terms using GOStat in the mRNA-seq data set (based on disease status, P≤0.05 and ≥±2.0 fold change, 708 genes).

GO ID	GO category	P-value	# Genes/ GO ID
278	mitotic cell cycle	3.98×10^−53^	44
279	M phase	5.26×10^−52^	42
22403	cell cycle phase	6.64×10^−48^	45
22402	cell cycle process	1.37×10^−42^	58
7049	cell cycle	4.51×10^−41^	68
48856	anatomical structure development	2.50×10^−30^	105
8283	cell proliferation	1.05×10^−29^	56
7275	multicellular organismal development	5.74×10^−29^	113
32502	developmental process	5.74×10^−29^	145
32501	multicellular organismal process	5.72×10^−28^	157
48731	multicellular systemic organismal development	9.09×10^−26^	86
48513	organ development	1.38×10^−24^	68
87	M phase of mitotic cell cycle	2.21×10^−24^	40
7067	mitosis	8.97×10^−24^	39
51301	cell division	2.17×10^−23^	39
65007	biological regulation	2.27×10^−18^	211
50789	regulation of biological process	1.37×10^−14^	188
74	regulation of progression through cell cycle	3.22×10^−14^	27
48869	cellular developmental process	6.84×10^−14^	77
30154	cell differentiation	6.84×10^−14^	77

We also evaluated the top GO categories in ECO-affected cells versus unaffected cells using KEGG pathway analysis for the microarray and mRNA-seq data sets (Tables 5 and 6, respectively). Interestingly, although several overrepresented pathways were the same using data from each platform, the most significant pathway - found from the mRNA-seq data - was the cell cycle. Overall, KEGG pathway analysis suggested downstream transcriptional consequences of the germline *ICK* mutation affect JAK-STAT and Wnt signalling pathways, cell adhesion and cytoskeletal structure, consistent with a role in regulation of cell proliferation.

**Table 5:** Top 20 overrepresented KEGG pathways in the cDNA microarray data set (based on disease status, P≤0.05 and ≥±2.0 fold change, 206 genes).

Pathway name	# input genes in pathway	% input genes in pathway	P-value
Jak-STAT signaling pathway^a^	9	4.39	4.46×10^−5^
Cell adhesion molecules (CAMs)^a^	8	3.902	1.10×10^−4^
Wnt signaling pathway	8	3.902	3.71×10^−4^
Focal adhesion^a^	7	3.415	7.77×10^−3^
Melanoma^a^	4	1.951	8.89×10^−3^
Regulation of actin cytoskeleton^a^	7	3.415	0.0101
Hematopoietic cell lineage	4	1.951	0.0147
Colorectal cancer	4	1.951	0.0166
TGF-beta signaling pathway^a^	4	1.951	0.0180
Prostate cancer	4	1.951	0.0210
Cytokine-cytokine receptor interaction	7	3.415	0.0238
Vibrio cholerae infection	3	1.463	0.0270
Acute myeloid leukemia	3	1.463	0.0296
Pathways in cancer^a^	8	3.902	0.0355
Systemic lupus erythematosus	4	1.951	0.0422
p53 signaling pathway^a^	3	1.463	0.0445
Thyroid cancer	2	0.976	0.0455
Complement and coagulation cascades^a^	3	1.463	0.0461
Antigen processing and presentation	3	1.463	0.0478

a pathways found in both platforms.

**Table 6:** Top 20 overrepresented KEGG pathways from the mRNA-seq data set (based on disease status, P≤0.05 and ≥±2.0 fold change, 708 genes).

Pathway Name	# input genes in pathway	% input genes in pathway	P-value
Cell cycle	18	2.542	2.68×10^−8^
Jak-STAT signaling pathway^a^	12	1.695	5.37×10^−3^
Pathways in cancer^a^	20	2.825	6.06×10^−3^
p53 signaling pathway^a^	7	0.989	6.99×10^−3^
Cell adhesion molecules (CAMs)^a^	10	1.412	0.0122
Renin-angiotensin system^a^	3	0.424	0.0172
TGF-beta signaling pathway^a^	7	0.989	0.0234
Regulation of actin cytoskeleton^a^	13	1.836	0.0240
Complement and coagulation cascades^a^	6	0.847	0.0261
Primary immunodeficiency	4	0.565	0.0273
Melanoma^a^	6	0.847	0.0295
Pancreatic cancer	6	0.847	0.0313
Focal adhesion^a^	12	1.695	0.0328
Gap junction	7	0.989	0.0376

a pathways found in both platforms.

## Discussion

We used two different methods, namely cDNA microarrays and the mRNA deep sequencing platform, to profile transcriptomes of fibroblasts from patients with ECO syndrome due to the homozygous R272Q mutation in *ICK*. We identified a hyperproliferative phenotype for cultured ECO cells and showed differential expression of genes involved in cell growth and proliferation. We also had a unique opportunity to compare the findings of these two platforms. We found that the mRNA-seq platform was more sensitive in identifying significantly differentially expressed genes than the cDNA microarray platform. Also, correlation with qRT-PCR validation experiments of fold-changes with mRNA-seq was also superior compared to cDNA microarrays. It is interesting to note that results from cDNA microarray and qRT-PCR do not correlate well for the top 20 genes acquired from the mRNA-seq platform data, indicating that although qRT-PCR shows biological differences for these genes, their changes in expression were not appreciated using the cDNA microarray. This also implies that the cDNA microarray platform contains numerous false-negatives, which may lead to inaccurate conclusions about the transcripts expressed in cases versus controls.

Initially transcription profiling studies largely relied on hybridization-based technologies. However with the introduction of mRNA-seq technology, RNA analysis through deep sequencing is achievable on a massive scale. Although the discussion of the advances and challenges of both platforms used here is beyond the scope of this paper, we will briefly address them. The microarray-based approach to study gene expression is high throughput and relatively inexpensive; however it has a limited range of detection due to both background and saturation of signals [17] and seems largely limited in its ability to catalogue and quantify diverse RNA molecules due to the reliance on probes for pre-specified targets [18]. mRNA-seq technology, on the other hand, has highly reproducible results with relatively little technical variation and has the potential to detect and quantify RNAs with low and moderate abundance since this approach digitally counts sequence reads [19]. However, by using sequence reads for RNA quantification, other issues arise; for instance, a small number of very highly expressed genes (7%) accounts for most of the reads (75%) [20]. More specifically in this study RPKM (Reads Per Kilobase of exon model per Million mapped reads), was the unit of measurement used to quantify transcript abundance. However, this unit is biased towards larger genes and ignores the fact that that different isoforms of a gene may be of different lengths [14].

Recently, others have compared the results of deep sequencing- and microarray-based transcriptional profiling in a mouse model of cardiomyopathy [21]. As we have now shown with our human transcriptome findings, those authors similarly concluded that mRNA-seq was sensitive and reliable in quantifying lower-abundance genes, which represented the majority of the regulated genes in their model.

We note that skin fibroblasts of the affected individuals were hyperproliferative in culture compared to those from normal individuals, which was consistent with the presumed role of ICK as a human cyclin-dependent kinase 2 (CDK2) member of the MAPK family. MAPKs are regulators of cell cycle and thus of cellular proliferation and apoptosis [22]. The expression experiments, as well as such clinical manifestations of ECO as cleft lip and palate, polydactyly, and dysplastic organs, support a role for ICK as a regulator of cell growth. Functionally, GO analysis showed some overlap between microarray and mRNA-seq data with respect to overrepresented pathways in cells from ECO patients. However, emphasis on biological pathway involvement is based on platform selection, such that cDNA microarray concentrates on pathways with phenotypic relevance to the disorder, while the mRNA-seq platform identifies a higher proportion of upstream genes involved in cell division and DNA replication pathways. It would be of interest to examine transcription profiles in cells of other types and from other tissues in ECO patients. Thus, mRNA-seq discovered more differentially expressed genes and showed better correlation with qRT-PCR than did microarrays in cultured skin fibroblasts from ECO patients. Because of the growing use and accessibility of new genomic technologies for clinical applications, the findings show that results should be carefully interpreted, since different methods can generate very different hypotheses. Further, the findings emphasize the importance of validation of high-throughput genome-wide approaches using an independent method, such as qRT-PCR.
